# Nonextraction Treatment of Severe Crowding with the Aid of Corticotomy-Assisted Orthodontics

**DOI:** 10.1155/2012/694527

**Published:** 2012-07-17

**Authors:** Ali S. Aljhani, Khalid H. Zawawi

**Affiliations:** ^1^Division of Orthodontics, College of Dentistry, King Saud University for Health Sciences and National Guard Health Affairs, P.O. Box 22490, Riyadh, Saudi Arabia; ^2^Division of Orthodontics, Faculty of Dentistry, King Abdulaziz University, P.O. Box 80209, Jeddah 21589, Saudi Arabia

## Abstract

This paper illustrates the combined nonextraction orthodontic treatment with the corticotomy technique in an adult patient (age: 25 years and 3 months) with severely crowded arches to accelerate tooth movement and shorten the treatment time. Both her upper lateral incisors were congenitally absent and both upper central incisors' roots were short. Initial fixed orthodontic appliances (bidimensional) were bonded and one week later buccal and lingual corticotomy with alveolar augmentation procedure in the maxilla and mandible was performed. Orthodontic activation to level and align and unravel the crowding was performed every two weeks. The total treatment time was 8 months with no adverse effects observed at the end of active treatment. The addition of the decortication procedure to the conventional orthodontic therapy decreased the duration of treatment significantly. Successful alignment of both arches with ideal overbite and overjet as well as adequate occlusion was achieved.

## 1. Introduction

An increasing number of adults in recent years are undergoing orthodontic treatment. Correction of malocclusion makes it possible to improve their periodontal health and psychosocial status [1]. It was shown in the Medical Expenditure Panel Survey (MEPS) that orthodontic visits by adults were 23.1% of all orthodontic visits [2]. This was also demonstrated in the fifth study of orthodontic and treatment procedures in 2008 that the percentage of adult active patients was about 20% of the total patients being treated in orthodontic offices in the United States [3]. The duration and management of comprehensive orthodontic treatment for adults is significantly longer compared to adolescent patients [4, 5]. Treatment modifications have been suggested to reduce the treatment time. The technique to accelerate tooth movement was first introduced by Köle, in 1959 [6]. This technique involved a radicular corticotomy and supra-apical osteotomy. The theory was that by creating blocks of bone with vertical buccal and lingual corticotomies and supra-apical horizontal osteotomy connecting cuts, segments of bone with the embedded teeth could be moved rapidly. Later, Anholm and coworkers [7] completed a nonextraction treatment of a case with severe malocclusion in 11 months using corticotomy. Suya [8] suggested modification to the osteotomy cuts with horizontal corticotomy. Gantes et al. [9] presented orthodontic cases treated with corticotomy in which they performed circumscribing corticotomy cuts both facially and lingually around the six upper anterior teeth. The upper first bicuspids were removed and the bone over the extraction sockets was removed both buccally and lingually. Chung et al. [10] removed blocks of medullary bone, block by block, with orthopedic forces. Similarly, Hwang and Lee [11] performed intrusion of unopposed molars by performing a corticotomy procedure and used magnets to deliver the force.

The corticotomy technique was later modified by Wilcko et al. with the addition of alveolar augmentation with resorbable, alloplastic freeze-dried bone to increase the volume of alveolar bone, regenerate bone affected by possible dehiscence and fenestration, and prevent gingival recession. Their justification was that the movement does not result from repositioning of tooth-bone blocks, but rather from a cascade of transient localized reactions in the bony alveolar housing leading to bone healing [12]. Since then, limited numbers of cases treated with corticotomy were reported in the literature. Germeç et al. [13] reported the treatment of an adult patient with a modified corticotomy technique, in which the lingual vertical and subapical horizontal cuts were eliminated, for the retraction of the lower anterior teeth after the extraction of four premolars. In a preliminary study, Fischer [14] compared the movement of impacted canines after surgical exposure using conventional surgical technique with their contralateral canines exposed using a corticotomy-assisted technique, and he concluded that corticotomy-assisted surgical technique reduced the orthodontic treatment time by 28–33%.

This paper illustrates in detail the effect of rapid orthodontic treatment of a severely crowded case with compromised upper central incisors with the aid of decortication technique and to discuss the surgical and the orthodontic steps involved. 

## 2. Case Report

A female patient (age: 25 years and 3 months) presented with a chief complaint of: *“I want my teeth fixed quickly”*. Her medical was noncontributory and she presented with several restorations, extractions of third molars, and good oral hygiene. 

### 2.1. Pretreatment Evaluation

**Figure 1:**
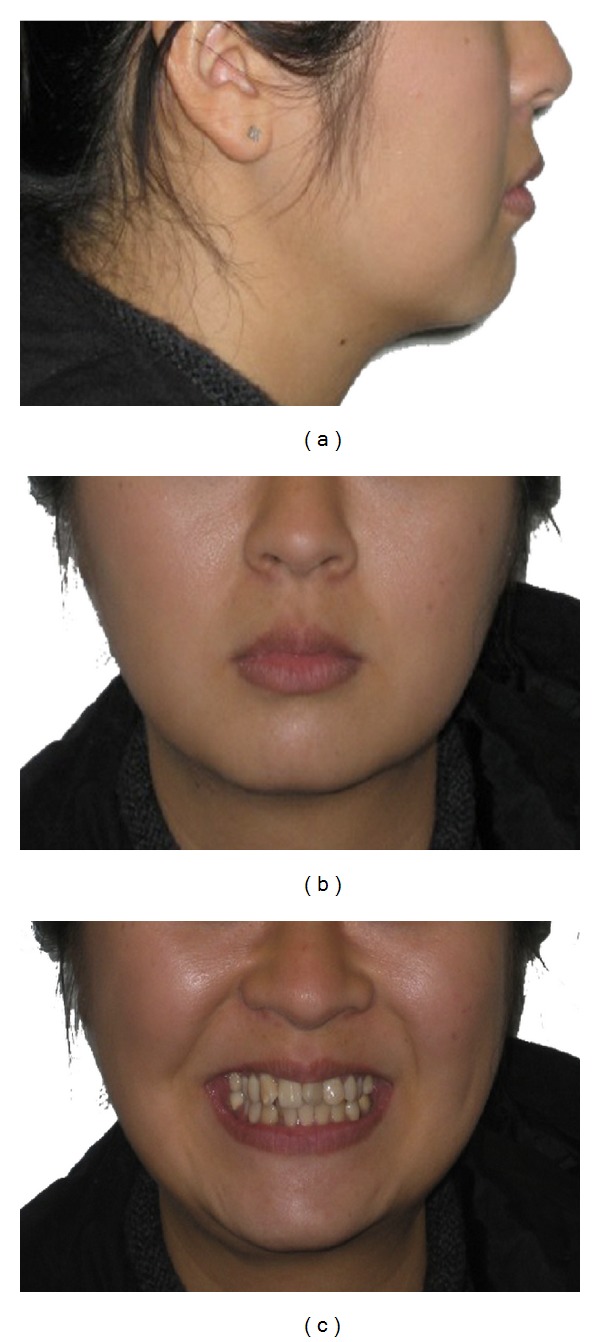
Pretreatment extraoral photographs.

The patient had a mild convex profile with mild protrusive and competent lips and shows 75% of her upper teeth when smiling. Nasolabial angle was within normal limits (Figure 1).

**Figure 2:**
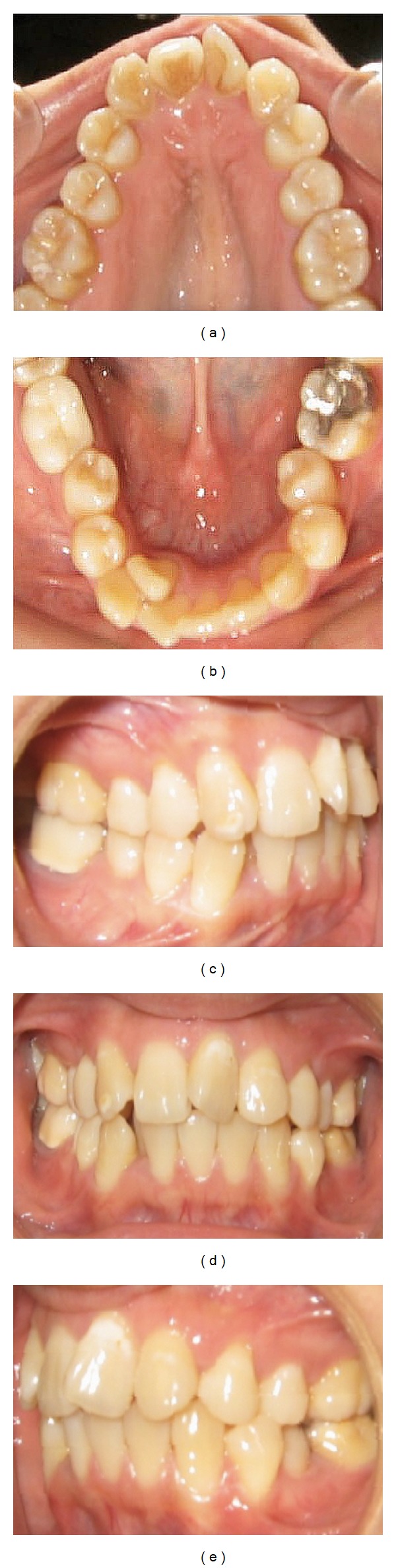
Pretreatment intraoral photographs.

Dental analysis revealed a full cups class II molars and canines relationships with 4-5 mm overjet and 2 mm overbite; maxillary arch was mildly crowded (4 mm) and mandibular arch was severely crowded (8-9 mm) with the right lateral incisor lingually crowded. Her upper right and left lateral incisors were missing. Upper midline was deviated to the right 2 mm and lower was deviated 4 mm to the right (Figure 2). 

**Figure 3:**
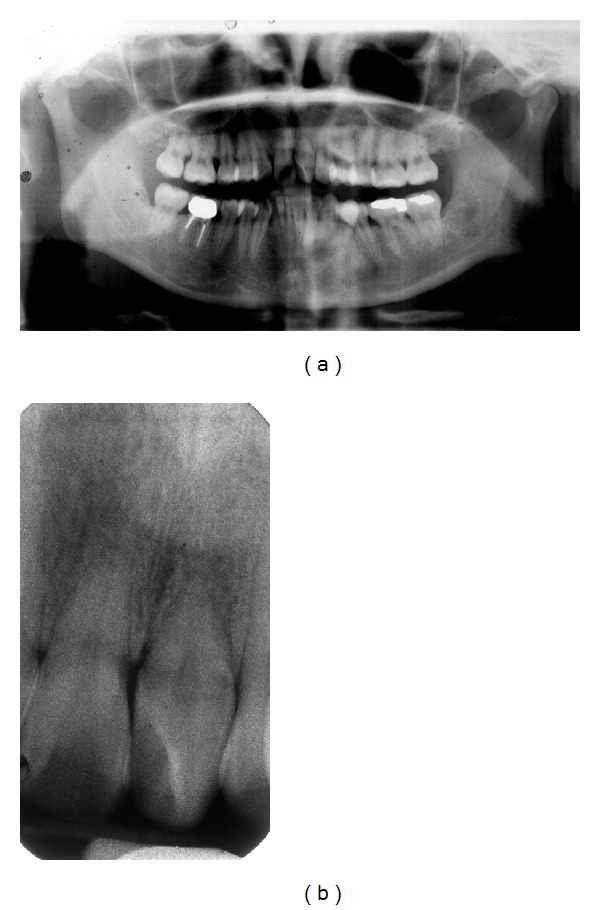
Pretreatment panoramic and periapical radiographs.

The panoramic radiograph confirmed the absence of the lateral incisors and showed the existence of short rooted upper right and left central incisors with no pathology. The lower left first molar had a large occlusodistal amalgam restoration. This was confirmed with a periapical radiograph (Figure 3).

**Figure 4:**
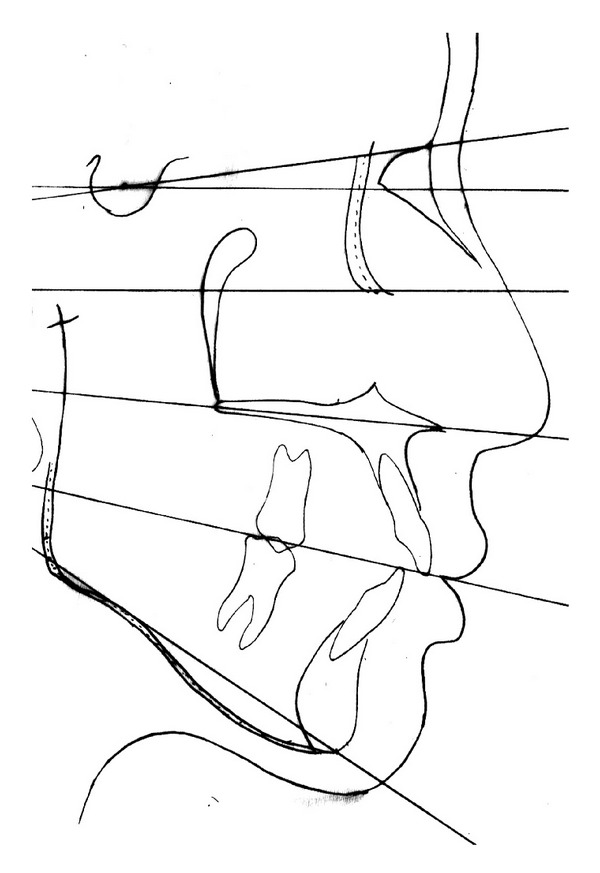
Pretreatment cephalometric tracing.

Cephalometric measurements showed a skeletal class I with hyperdivergent mandibular plane with moderately proclined and protrusive lower incisors (Table 1 and Figure 4).

### 2.2. Treatment Plan

The treatment options were discussed with the patient. The initial option of lower premolar extraction to relief the crowding, align the lower arch, and control possible bite opening during treatment due to the hyperdivergent mandibular plane angle. The patient declined this option and showed a genuine interest to have the orthodontic treatment completed as soon as possible and she was not willing to extract any teeth to relieve the crowding. The treatment plan involved the following comprehensive orthodontic treatment with fixed appliances to align the upper and lower teeth and correct the crowding and corticotomy procedure in both arches to accelerate the tooth movement. Due to the congenitally missing upper lateral incisors and lack of space the canines will substitute the missing laterals and first premolars will substitute the canines and finish in class II molar relationship. Also, the patient was informed that the lower left first molar large amalgam restoration could be compromised during treatment.

### 2.3. Treatment Progress

One week before the surgery, fixed appliances (Clarity, 3 M Unitek, USA, Bi-Dimensional prescription appliances, that is, 0.018 × 0.025-inch slot brackets for the incisors and 0.022 × 0.028-inch for the canines and the posterior teeth) and 0.016-inch NiTi arch wires were inserted for initial leveling and alignment. The lower right lateral incisor was not engaged.

### 2.4. Surgical Procedure

**Figure 5:**
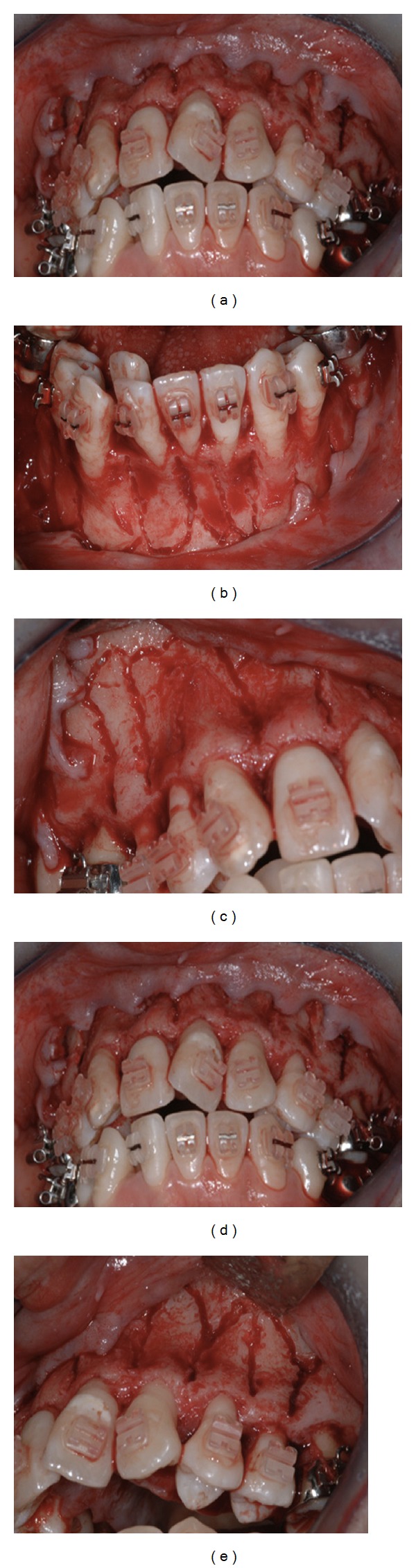
Maxillary and mandibular corticotomy of the buccal side.

Corticotomy technique as described by Wilcko [12] was performed by a periodontist. Briefly, after administering the proper anesthetic dose, a full thickness flap was reflected sharply both facially and lingually around all erupted teeth, in both arches, from the first molar to the first molar (Figure 5). Each flap was released with a sulcular incision and with papillary preservation technique when possible. The lingual interdental papilla between the maxillary central incisors was not reflected and no vertical releasing incisions were used. Cuts in the alveolus that penetrate the entire thickness of the cortical plate and penetrate just barely into the medullary bone were performed both buccally and lingually around all the teeth in both arches. Vertical decortication cuts were made between the roots of the teeth and they were stopped 2-3 mm shy of the alveolar crest. Horizontal scalloped corticotomy cuts were used to connect the vertical cuts along with perforations in the cortical plate. Bone grafting mixture OraGraft demineralized cortical particulate (LifeNet Health, Inc., Virginia Beach, VA, USA) was then applied to the activated cortical plates. Flaps were repositioned to their pre-surgical positions and sutured with interrupted loop sutures. Nonresorbable sutures (Gore-tex, CV-5, RT-16, from W. L. Gore & Associates, Inc., Medical Products Division, Flagstaff, AZ, USA) were used and the sutures were removed after 14 days from the procedure. The patient was kept under antibiotic for 10 days following the surgery.

**Figure 6:**
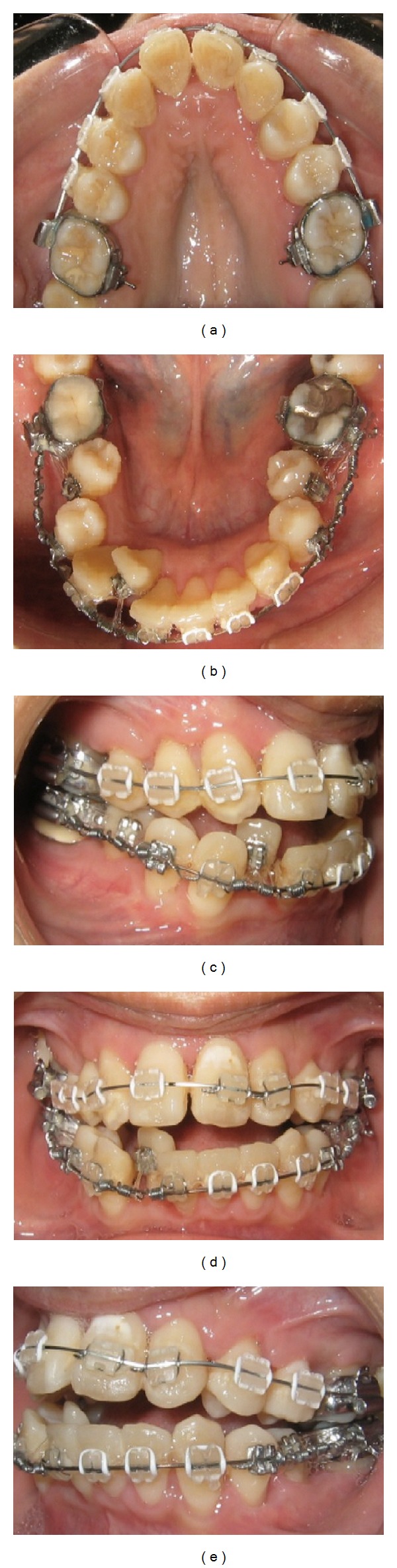
Intraoral photographs 2 weeks after corticotomy.

At the suture removal appointment, 0.016 × 0.022-inch NiTi archwire was inserted in the upper arch and 0.016-inch stainless steel was inserted in the lower arch with 150 g NiTi push coil springs to create space for the ectopic lower right lateral incisor (Figure 6).

**Figure 7:**
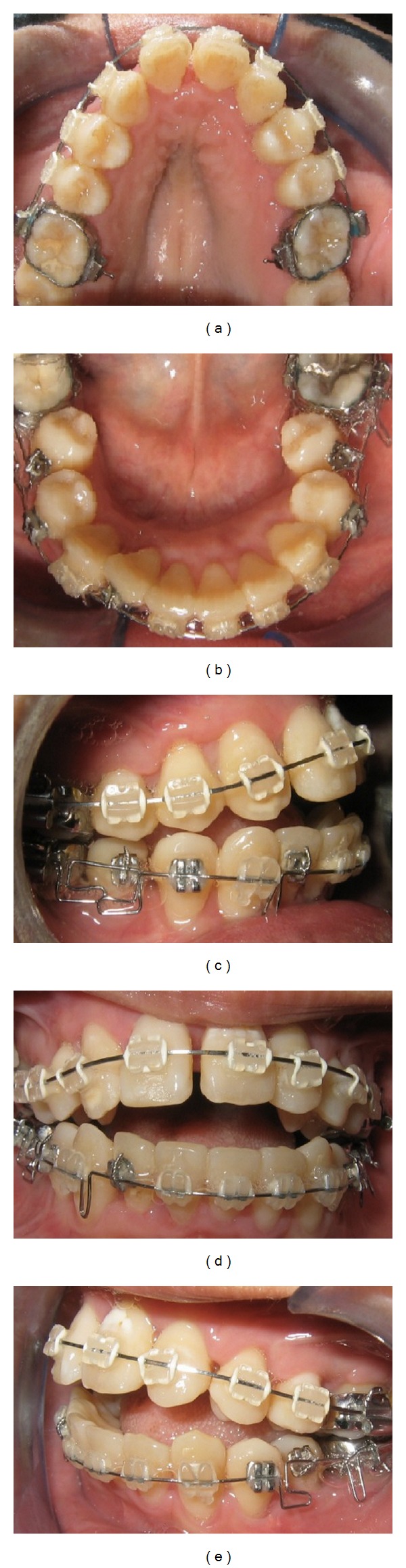
Intraoral photographs 10 weeks after corticotomy.

**Figure 8:**
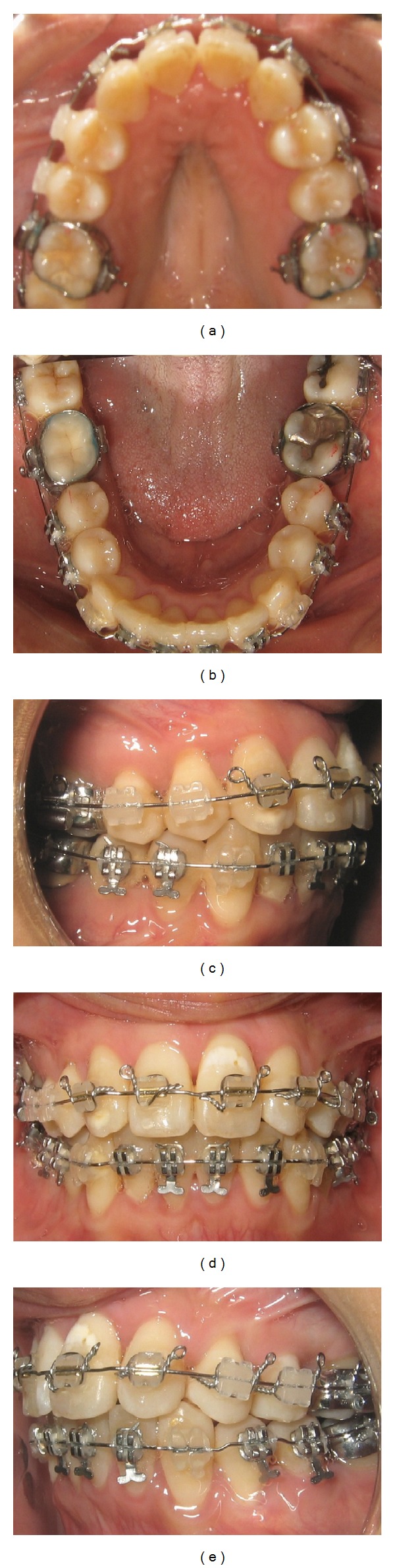
Intraoral photographs 28 weeks after corticotomy.

After 10 weeks, postoperatively, the ectopic lateral incisor was bonded and engaged with a power thread. The patient developed an open bite that was anticipated due to her hyperdivergent mandibular plane and as her lower premolars started to align. Vertical elastics were used to control for the open bite (Figure 7). She was instructed to wear Class II box elastics (“3/16”, 4 oz) full-time. The patient was extremely compliant and used the elastic as instructed. During the 28th week postoperatively, the upper and lower arched were leveled and aligned, the ectopic lower right lateral incisor was in proper position (Figure 8).

**Figure 9:**
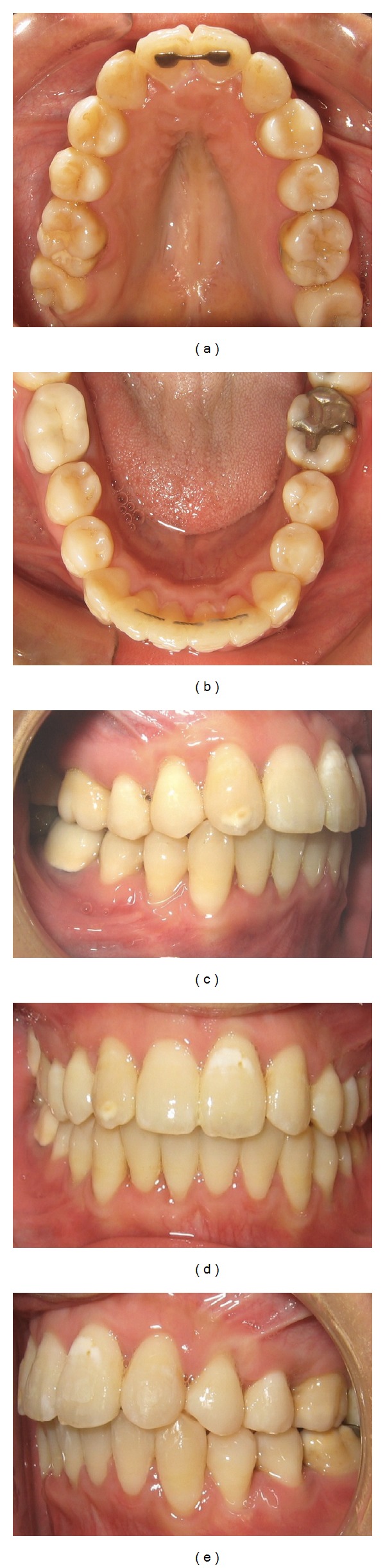
Intraoral photographs 32 weeks after corticotomy.

After 33 weeks of active treatment, the upper and lower crowding and rotations were corrected. The teeth are well aligned with good intercuspal relation. The total treatment period with the fixed appliance therapy was 8 months. After the appliance was removed, lingual bonded retainers were set on both the upper and lower arches and removable upper circumferential and lower Hawley retainers were delivered to be worn full time for the first 6 months (Figure 9).

### 2.5. Treatment Results

**Figure 10:**
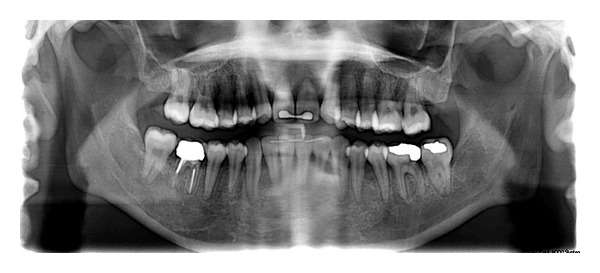
Posttreatment panoramic radiograph.

Post-treatment evaluation of the patient revealed a normal overjet and overbite, upper and lower midlines were coincident to one another and to the facial midline. The upper and lower dental arches are well aligned and class II molars and canines relationships was achieved as planned. Also, good preservation of the interdental papillae with no gingival recession was evident. Posttreatment panoramic radiograph showed good parallelism of the roots, no significant reduction in the radiographic height of the crestal bone, and no evidence of any significant apical root resorption. Furthermore, the roots of the upper central incisors condition was stable (Figure 10).

**Figure 11:**
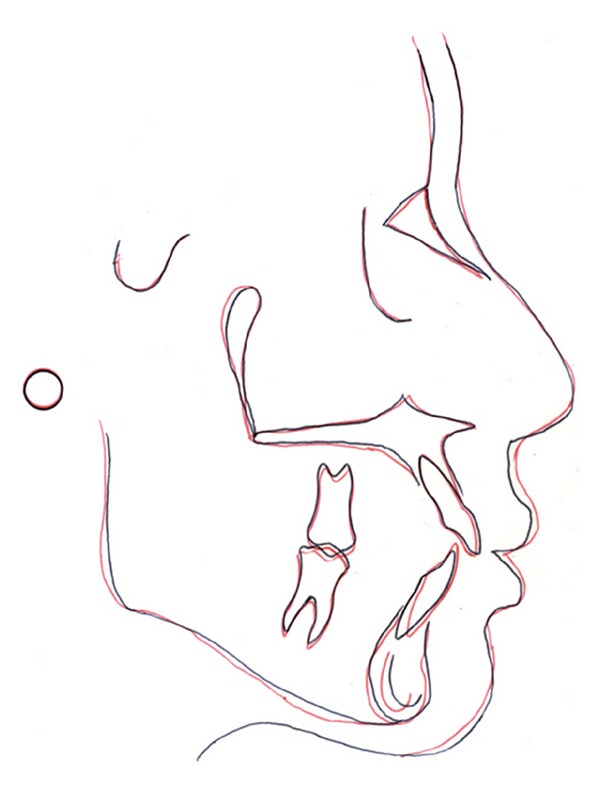
Cephalometric superimposition (pretreatment: black and posttreatment: red).

**Figure 12:**
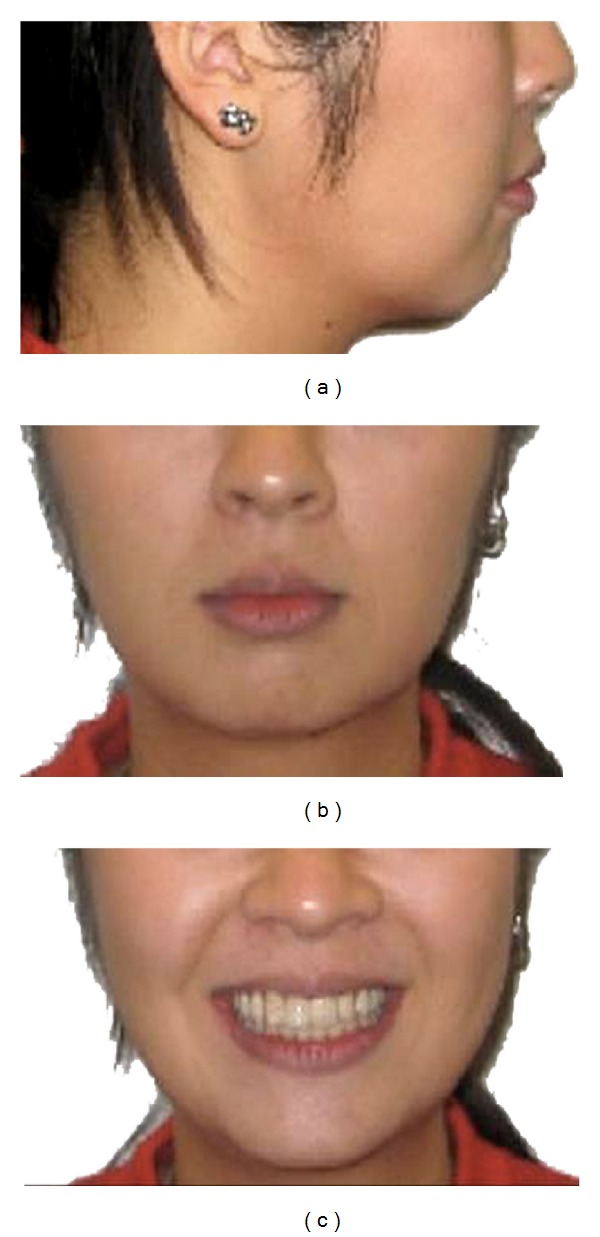
Posttreatment extraoral photographs.

Final lateral cephalometric analysis showed minimal proclination of the upper and lower anterior teeth with mild upper and lower lip protrusion (Table 1 and Figure 11). Eventhough the nasolabial angle decreased, it remained within normal limits (Figure 12).

## 3. Discussion

The comprehensive orthodontic treatment of this adult patient who presented with severe lower crowding was completed in 8 months which is significantly less than the routine duration that conventional orthodontic treatment of similar cases require. The addition of the corticotomy procedure has been reported to shorten the conventional orthodontic treatment time and it was claimed that teeth can be moved 2 to 3 times further in 25 to 30% less time required for traditional orthodontic treatment [7–11, 15, 16]. The current paper confirms previously published findings and supports this treatment option. 

The concept of corticotomy as initially introduced and later adapted by several investigators relied on creating blocks of bone with the embedded teeth that can be moved rapidly with heavy forces [6–10]. On the other hand, conventional orthodontic forces were advocated by Wilcko et al. [12, 15, 16] who explained the rapid tooth movement as an illustration of regional acceleratory phenomenon (RAP). Frost described this process in 1983, and found that the surgical wounding of osseous tissues results in accelerated regional healing process. It can accelerate both hard and soft tissue healing 2 to 10 folds and this leads to decreased regional bone density and accelerated bone turnover [17–19]. This was reported to occur also in intraoral cortical bone by others [20–22]. Wilcko and coworkers showed that transient localized de- and remineralization in the bony alveolar housing occurs and the demineralization of the alveolar bone over the root surfaces leaves the collagenous soft tissue matrix of the bone, which can be carried with the root surface and then remineralizes following the completion of the orthodontic treatment [15]. Initially, the term “Accelerated Osteogenic Orthodontics” was used to describe the combined corticotomy-facilitated orthodontics and periodontal alveolar augmentation [12]. Later, Wilcko et al. [15] modified it to be *“Periodontally Accelerated Osteogenic Orthodontics”* (PAOO) technique. In a recent report comparing the alveolar bone reactions to osteotomy-assisted and corticotomy-assisted tooth movement, Lee et al. [23] found evidence of regional accelerated phenomenon in the alveolar bone of the corticotomy-treated animals and distraction osteogenesis in the osteotomy-assisted tooth movement using micro-Computed Tomography. This confirms the hypothesis of the difference in the healing processes involved in both procedures. 

Original corticotomy-facilitated orthodontic treatment involved buccal and lingual osteotomy cuts with orthopedic forces, and the use of alveolar augmentation with demineralized bone graft was advocated to cover any fenestration and dehiscence and to increase in the bony support for both the teeth and the overlying and soft tissues [12, 16]. Surface computed tomographic scans taken before and after the treatment showed a return of the layer of mineralized bone over the roots of the teeth and a general increase of the bone volume [12, 16]. More controlled studies are still warranted to investigate the importance of the alveolar augmentation in the decortication technique and the effects of different grafting materials on the alveolar bone. While osteotomy cuts were recommended on both alveolar surfaces (buccal and lingual) [6, 12, 16], two recently published case reports showed the results of selective corticotomy limited to the buccal and labial surfaces to reduce the operation time and postoperative patient discomfort and avoid the risk of violating vital lingual anatomy [13, 24]. The current case was managed with both buccal and lingual cuts and efficient tooth movement was observed, however, elimination of the lingual cuts should be studied in future trials.

Most of the recently published literature showed no adverse effects of the corticotomy on the periodontium [13, 15, 24–26]. In the present case, no reduction in the crest bone height, gingival recession, and apical root resorption was seen after the orthodontic treatment. The upper central incisors did not exhibit any further resorption and were stable during the treatment. The disadvantages of this procedure are the need for more frequent activation compared to conventional orthodontic treatment, the extra expenses for the periodontal surgery, and the postoperative discomfort were the disadvantages of this technique that the patient need to tolerate for a faster course of treatment.

Since most of what is known about the decortication procedures in orthodontic treatment is based on case reports, more laboratory and clinical studies are necessary to better explain the biological mechanisms involved at both the tissue and molecular level, and most importantly, investigate the long term effects of this procedure. 

## 4. Conclusions

Comprehensive orthodontic treatment with the aid of the decortication technique is an effective treatment option in adults to achieve the desired results in significantly reduced treatment duration. 

## Figures and Tables

**Table 1:** Summary of cephalometric measurements.

Measurement	Norm	Before treatment	After treatment
SNA (degrees)	82	78	78
SNB (degrees)	80	75	74
ANB (degrees)	2	3	4
Wits appraisal (mm)	0	1	1
FMA (degrees)	25	36	35
U1-NA (degrees)	22	22	24
U1-NA (mm)	4	6	7
L1-NB (degrees)	25	30	31
L1-NB (mm)	4	7	7
IMPA (degrees)	90	94	97
Nasolabial angle (degrees)	102	98	94
E-Line-Upper lip (mm)	−4	−2	0
E-Line-Lower lip (mm)	−2	0	2
